# Redox control of protein degradation

**DOI:** 10.1016/j.redox.2015.07.003

**Published:** 2015-09-09

**Authors:** Marta Pajares, Natalia Jiménez-Moreno, Irundika H.K. Dias, Bilge Debelec, Milica Vucetic, Kari E. Fladmark, Huveyda Basaga, Samo Ribaric, Irina Milisav, Antonio Cuadrado

**Affiliations:** a Instituto de Investigaciones Biomédicas Alberto Sols (CSIC-UAM), 28029 Madrid, Spain; b Instituto de Investigación Sanitaria La Paz (IdiPAZ), 28029 Madrid, Spain; c Centro de Investigación Biomédica en Red de Enfermedades Neurodegenerativas (CIBERNED), ISCIII, Spain; d Aston Research Centre for Healthy Ageing, Life and Health Sciences and, Aston University, Aston Triangle, Birmingham B4 7ET, UK; e Department of Pharmaceutical Biotechnology, Faculty of Pharmacy, Ege University, 35100 Bornova, Izmir, Turkey; f Department of Physiology, Institute for Biological Research "Sinisa Stankovic", University of Belgrade, 11060 Belgrade, Serbia; g Department of Molecular Biology, University of Bergen, Bergen N-5020, Norway; h Molecular Biology Genetics and Bioengineering Preogram, Sabanci University, 34956 Tuzla, Istanbul, Turkey; i University of Ljubljana, Faculty of Medicine, Inst. of Pathophysiology, Zaloska 4, SI-1000 Ljubljana, Slovenia; j Laboratory of Oxidative Stress Research, Faculty of Health Sciences, Zdravstvena pot 5, SI-1000 Ljubljana, Slovenia

**Keywords:** Aβ, amyloid beta, AMPK, AMP activated protein kinase, ASK1, apoptosis signal-regulating kinase 1, ATG4, autophagy related protein 4, ATM, ataxia-telangiectasia mutated, BCL-2, B-cell lymphoma 2, CMA, chaperone mediated autophagy, ER, endoplasmic reticulum, GSH, reduced glutathione, GSSG, oxidized glutathione, HIF-1, hypoxia inducible factor-1, IKKB, inhibitor of nuclear factor kappa-B kinase subunit beta, JNK1, c-Jun N-terminal kinase, LC3, microtubule-associated protein light chain 3, NFκB, nuclear factor kappa B, NOX, nicotinamide adenine dinucleotide phosphate oxidase, NRF1/2, nuclear factor (erythroid-derived 2)-like 1/2, PARP1, poly [ADP-ribose] polymerase 1, PDH, prolyl-4-hydroxylase, PDIs, protein disulfide isomerase, PI3K, phosphatidylinositol 3-kinase, PrP, prion protein (PrPc, cellular form, PrPsc, scrapie form), PTEN, phosphatase and tensin homolog, ROS, reactive oxygen species (mtROS, mitochondrial ROS), α-SYN, α-synuclein, mTORC1, mammalian target of rapamycin complex 1, Trx, thioredoxin, TSC2, tuberous sclerosis complex 2, Ub, ubiquitin, ULK1, unc-51 like autophagy activating kinase 1, UPR, unfolded protein response, UPS, ubiquitin proteasome system, Protein oxidation, Proteolysis, Proteasome, Autophagy, Unfolded protein response

## Abstract

Intracellular proteolysis is critical to maintain timely degradation of altered proteins including oxidized proteins. This review attempts to summarize the most relevant findings about oxidant protein modification, as well as the impact of reactive oxygen species on the proteolytic systems that regulate cell response to an oxidant environment: the ubiquitin-proteasome system (UPS), autophagy and the unfolded protein response (UPR). In the presence of an oxidant environment, these systems are critical to ensure proteostasis and cell survival. An example of altered degradation of oxidized proteins in pathology is provided for neurodegenerative diseases. Future work will determine if protein oxidation is a valid target to combat proteinopathies.

## Highlights

• Proteins undergo reversible and irreversible redox modifications.
• Oxidized proteins are cleared mainly through the 20S proteasome and autophagy.
• The proteolytic systems exhibit a dynamic crosstalk to adapt to redox alterations.
• Protein oxidation together with impaired degradation are linked to neurodegeneration.

## Graphical abstract

**Figure:**
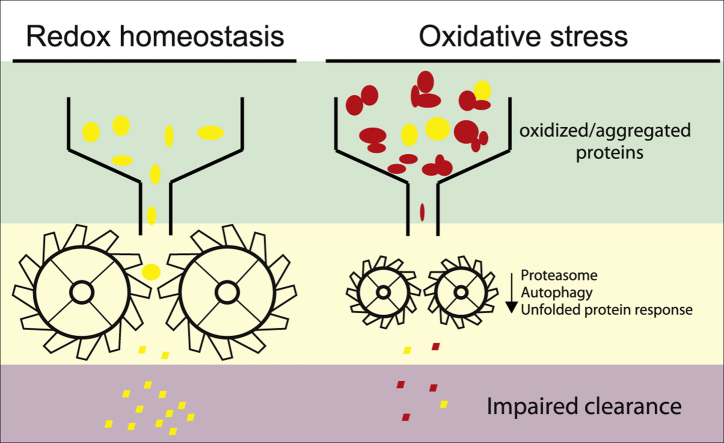

## 1 Introduction to redox homeostasis (redoxtasis)

Aerobic metabolism has the advantage of a better energy yield, but at the cost of generating reactive oxygen species (ROS). Indeed, the leakage of superoxide from mitochondrial respiratory chain complexes I and III constitutes one of the major sources of ROS production [1]. Other sources of harmful ROS include unfolded protein response at the endoplasmatic reticulum (ER) [2], and oxidant byproducts generated at peroxisomes [3,4]. Moreover, evolution has used oxygen to modify certain proteins, now termed redox switches, as a cell signaling mechanism in survival [5] and regeneration [6] among other pathways. This clever use of ROS is best exemplified by NADPH oxidases (NOX), situated in the plasma membrane, whose main role is to generate superoxide and ultimately hydrogen peroxide (H_2_O_2_) as second messengers [7].

**Fig. 1:**
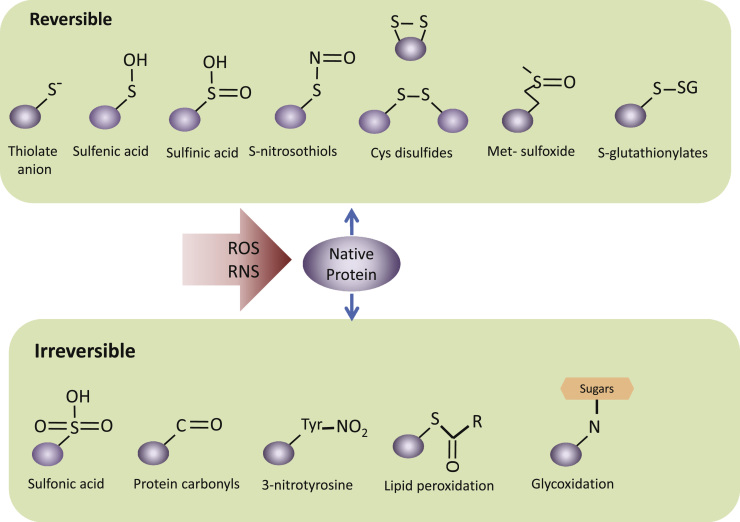
Oxidation of sulfur containing amino acids. A, Reversible modifications modulate physiological protein functions that act as molecular redox switches. The sulfur-containing amino acids methionine (Met) and cysteine (Cys) may undergo oxidation to generate Cys disulfides, S-thiolates, S-sulfenates, Met-sulfoxides, S-glutathionylates and S-nitrosothiols. B, Sulfur-containing amino acids can be further oxidized to irreversible sulfonic acid. Irreversible oxidative modifications, typically occur under the conditions of oxidative stress and lead to structural changes, protein inactivation and ultimately require protein degradation. Irreversible modifications lead to cleavage of the protein backbone by hydroxyl radical or direct oxidation or adduct formation of the side chain amino acids. Oxidative modifications to amino acids include the hydroxylation of aromatic groups and aliphatic amino-acid side chains, nitration of aromatic amino-acid residues, oxidized lipid adduction, conversion of amino-acid residues to carbonyl derivatives and glycoxidation (adduction of advanced aged glycation end products) (this figure has been adapted from [156] and [157]) .

Cells have efficient enzymatic and non-enzymatic strategies to modulate redox signaling and maintain redox homeostasis [8,9]. In addition, antioxidants are also obtained from exogenous sources, with the diet as the main supplier [10]. However, many pathological conditions or the normal decline in cell homeostasis related to ageing lead to a gradual imbalance between ROS formation and degradation and result in detrimental alterations of macromolecules. Sulfur containing amino acids, cysteine and methionine, are responsible for reversible and irreversible modification of proteins. In addition, proteins can form adducts with oxidizing byproducts. Fig. 1 summarizes oxidative modifications of sulfur containing amino acids.

In this review, we summarize the most relevant findings about the degradation of oxidized proteins, here termed oxyproteins, and the impact of oxidative stress on proteolytic cell systems, and gene expression.

## 2 The ubiquitin-proteasome system (UPS) in the control of oxyprotein degradation

The UPS participates in the degradation of soluble proteins in cytosol and nucleus [11]. The central core of the UPS is the 20S proteasome, which is present in animals, plants and bacteria. This 700 kDa-multisubunit protease is highly effective in the proteolytic degradation of misfolded or otherwise altered proteins. In eukaryotes, the 20S proteasome binds one or two regulatory 19S complexes to give rise to the 26S proteasome, changing its activity and specificity towards native folded proteins [12,13].

The main difference between ubiquitin (Ub)-dependent and Ub-independent proteasomal degradation is ATP-requirement. Based on this, the fact that ATP addition to cell lysate had no effect or even decreased proteasomal degradation of oxyproteins, led to the conclusion that these proteins are degraded by the 20S proteasome independently of both regulatory 19S complex and Ub [14–16]. In accordance to this, the 26S proteasome and the ubiquitinating machinery are much more sensitive than its 20S core to oxidative stress [17,18]. Thus, 20S proteasomal degradation is unaffected by H_2_O_2_ concentrations of up to 5 mM, while ATP-dependent degradation by the 26S proteasome begins to decline at 400 µM, and is completely abolished at 1 mM [19,20].

**Fig. 2:**
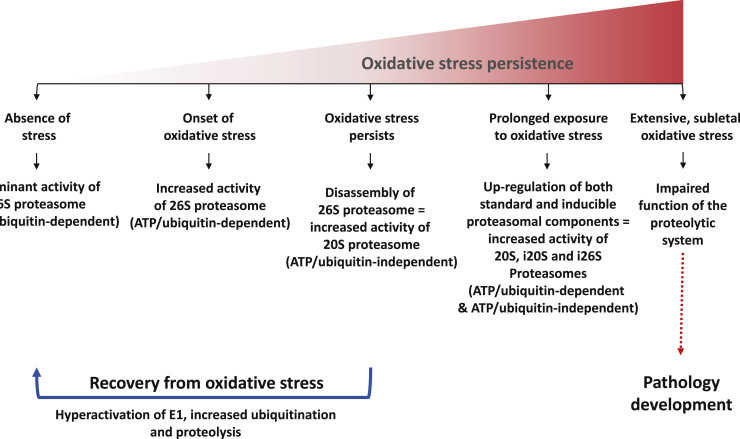
Proposed model of oxidative stress-dependent regulation of the 26S proteasome. Under basal conditions, 26S proteasome is the major cellular proteolytic machinery, which removes folded and functional proteins by ATP/Ub-dependent mechanism. It has been suggested that its activity may be enhanced upon the onset of oxidative stress, in order to protect the cell from oxidatively damaged proteins. However, under persistent oxidative stress conditions, the 26S proteasome disassembles, and the ubiquitinating system becomes deactivated. In this way 20S proteasome becomes the preferential cytosolyc, but also nuclear, proteolytic machinery. In accordance to this is the higher resistance of 20S proteasome to oxidative injures in comparison to its 26S counterpart. Released 20S proteasome may now degrade oxidatively degraded proteins in the ATP/Ub-independent manner. This is accomplished probably by recognition of exposed hydrophobic structures on the target protein, which are normally buried inside the natively folded protein, but become exposed as a consequence of oxidative modification-induced conformational changes of the proteins. Importantly, at this stage disassembly of 26S proteasome is reversible, and thus removal of oxidative stress leads to reassembly of the 26S proteasome, and recovery from oxidative injures. Furthermore, after several hours of recovery from oxidative stress, hyperactivation of the ubiquitinating system, as well as proteolysis, was observed. However, if oxidative stress continues proteasomal activities are inhibited and de novo synthesis of both standard and inducible proteasomal components (including 11S regulatory complex) is activated, giving rise to newly-formed 20S, i20S and i26S proteasomes, i.e. both ATP/Ub-dependent and-independent pathways of proteasomal degradation. If oxidative stress persist still and/or increases (sublethal level), the proteolytic system becomes impaired, giving rise to accumulations and aggregates of damaged or abnormal proteins.

On the other hand, some oxyproteins might require ubiquitin-dependent 26S proteasome degradation [21,22]. Thus, Dudek and co-workers [21] showed that carbonyl-containing proteins, a direct measure of protein oxidation, are selectively removed by ubiquitinating machinery. Furthermore, it has been reported that inhibition of USP14, deubiquitinating enzyme that associates with the proteasome, may enhance the clearance of oxyproteins, and thus, cellular resistance to oxidative challenges [22]. Based on the extensive review of Aiken and co-workers [23] and Shang and Taylor [24] changes in proteasomal activity upon oxidative pressure are illustrated in Fig. 2.

**Fig. 3:**
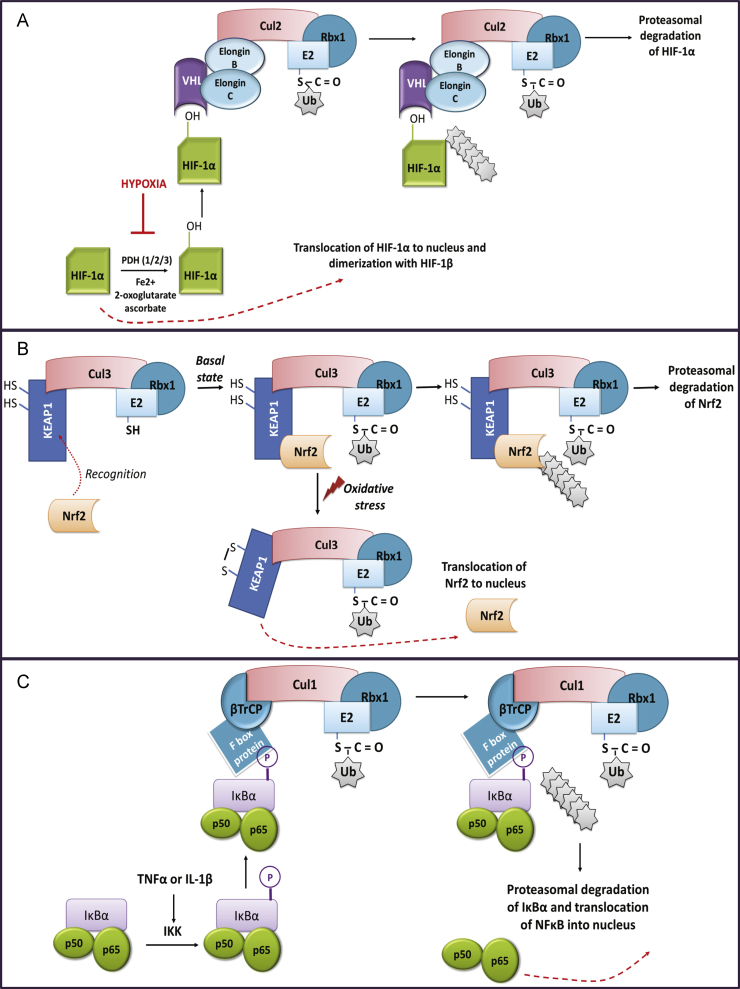
A. Regulation of the hypoxia-inducible transcription factor 1α (HIF-1α). HIF-1 is a heterodimer transcription factor that consists of an O_2_-sensitive α subunit and a constitutively expressed β subunit. The level of HIF-1α is determined by the relative ratio of its synthesis versus degradation. Under normoxic conditions HIF-1α is quickly degraded and exhibits a half-life of just about 5 min. HIF-1α is hydroxylated and rapidly degraded by the ubiquitin-proteasome pathway. HIF-1α stability is regulated via the activity of a class of oxygen-, 2-oxoglutarate-, and iron-dependent enzymes known as prolyl-4-hydroxylases (PDH), which hydroxylate two prolines at locations 402 or 564. Hydroxylation of HIF-1α created a recognition site VHL, a E3 ubiquitin ligase adapter recruits HIF-1α to the VHL-elongin B and C-Cul2 complex. Thus, VHL directs ubiquitination and subsequent proteasomal degradation of HIF-1α. By contrast, under oxygen-limiting conditions, HIF-1α is stabilized, and can translocate to the nucleus where it dimerizes with HIF-1β and activates transcription of genes containing hypoxia response elements (HREs). These genes include those that enhance hypoxia tolerance by increasing oxygen delivery to the tissues and anaerobic ATP-generation by glycolysis. B, Nuclear factor (erythroid-derived 2)-like 2 (Nrf2) is a master regulator transcription factor of redox homeostasis that functions in association with ‘small’ Maf proteins. Under basal redox conditions, NRF2 is localized in cytoplasm in association with Kelch ECH associating protein 1 (Keap1) and has a very short half life of about 20 min. The Keap1-Cul3-Rbx complex directs polyubiquitination of the Nrf2 and its subsequent proteasomal degradation. However, under oxidative or electrophilic conditions, specific Cys residues of Keap1 undergo oxidative modification. Bound NRF2 is not degraded and newly synthesized NRF2 is able to accumulate, translocate to the nucleus and induce the expression of target genes. In the nucleus Nrf2 drives the expression of genes containing an enhancer termed Antioxidant Response Element (ARE) such NAD(P)H quinone oxidoreductase 1, heme oxygenase 1, catalase, CuZn superoxide dismutase, glutamate-cysteine ligase, glutathione S-transferases, etc. C, The UPS plays also a role in activation of NFκB. According to canonical pathway of NFκB activation, ubiquitination and proteasomal degradation of its inhibitor (IκBα) releases NFκB, which now can freely translocate into nucleus. In this way, NFκB can activate transcription of genes vital for cellular response to stimuli such as stress, cytokines, free radicals, ultraviolet irradiation, and bacterial or viral antigens.

Apart from its role in the degradation of oxidatively damaged proteins, the proteasomal system is also involved in highly controlled degradation of proteins acting as redox switches. This is the case of certain transcription factors such as hypoxia-inducible factor-1 (HIF-1), Nuclear factor (erythroid-derived 2)-like 2 (NRF2) or nuclear factor kappa B (NFκB) [25,26] as illustrated in Fig. 3.

**Fig. 4:**
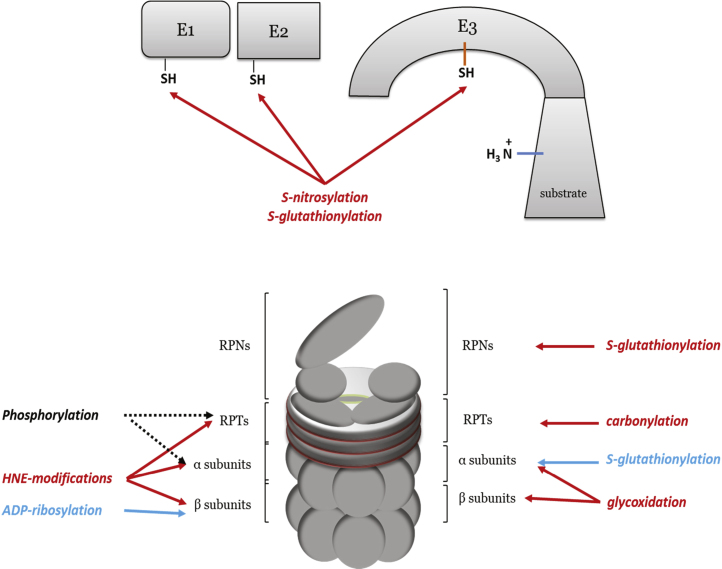
ROS-induced modification of the UPS. Red arrows suggest decreased activity of the proteasomal degradation upon induced modification, while blue arrows suggest that modification has opposite effects. On the other side, arrows that points ‘phosphorylation’ to the target proteasomal components are black because the effects of these modification is still experimentally unconfirmed, in the oxidative stress conditions. (For interpretation of the references to color in this figure legend, the reader is referred to the web version of this article.)

The UPS itself is subjected to alterations derived from oxidative stress. As shown in Fig. 4, ubiquitinating enzymes are inactivated by disulfide bond formation, S-nitrosylation, and S-glutathionylation [27,28]. The E3 ligase adapter kelch-like ECH-associated protein 1 (KEAP1) is an excellent example (Fig. 3B). The proteolytic activity of the proteasome requires intact sulfhydryl groups of Cys residues in the beta subunits of the calatytic 20S core for catalysis. These Cys are largely responsible for the susceptibility of the proteasome to oxidative insults. Moreover, both core 20S and regulatory 19S complexes are targets of oxidative modification, including 4-hydroxynonenal modification, carbonylation, S-glutathionylation, glycoxidation, as well as ADP-rybosilation and phosphorylation, which are indirect ROS-induced modifications (Fig. 4). Considering that the 26S proteasome is much more sensitive to oxidative stress than its 20S core, it is not surprising that almost all these modifications were detected on the 19S regulatory subunits leading to decreased activity of the 26S proteasome [29–31]. Although the majority of these modifications result in decreased 20S proteasome activity, some of them show the opposite effect. Thus, it has been shown that S-glutathionylation of two Cys residues on α5 subunits of 20S in yeast, increases proteasome activity via opening of the annulus [32]. Also, ADP-ribosylation of Glu, Asp, or Lys of nuclear 20S proteasome by poly[ADP-ribose] polymerase 1 (PARP1), increases its chymotrypsin-like activity [33]. The PARP1 is activated in the response to single-strand and double-strand breaks of DNA [34]. Besides ADP-ribosylation, phosphorylation seems to be important post-translation modification that may regulate proteasome activity upon oxidative stress [35,36], but this still needs to be experimentally confirmed.

Up-regulation of proteasome genes is coupled with enhanced capacity of the cell to cope with detrimental effects of oxidative insult [37]. Thus, it has been shown that increased expression of the UMP1, proteasome assembly protein, improves cell viability upon exposure to different oxidants, probably via up-regulation of the proteasome β subunits [38,39]. It is interesting that NFκB and activator protein-1 (AP1) do not seem to participate in regulation of most proteasome genes [40] while Nuclear factor (erythroid-derived 2)-like 1 (NRF1) and NRF2 appear to have a major role [40,41]. Thus, it seems that regulation of the proteasomal and NRF2 activity is bidirectional, i.e. in the basal conditions the proteasome down-regulates NRF2 activity by degradation, while upon oxidative stress, released NRF2 up-regulates proteasomal activity and protects the cell from the accumulation of oxyproteins. This effect may explain why pre-treatment of cultured neurons with low doses of proteasome inhibitors lead to increased, not decreased, proteasomal activity [42].

## 3 Autophagy and degradation of oxyproteins

Autophagy refers to any intracellular process that leads to degradation of cytosolic components inside lysosomes [43,44]. There are three different types of autophagy in mammals: macroautophagy, chaperone mediated autophagy (CMA) and microautophagy [44,45]. Macroautophagy (often referred to as autophagy) is a process in which a portion of the cytosol is surrounded by a growing double membrane (autophagosome) which eventually fuses with the lysosome (autophagolysosome), where the content will be degraded [43]. Cytosolic substrates with a KFERQ-like motif can be selectively recognized, translocated and degraded inside the lysosome by CMA [46,47]. Finally, the direct invagination of the lysosomal membrane can introduce cytosolic portions into the lysosome in a type of autophagy called microautophagy [48].

**Fig. 5:**
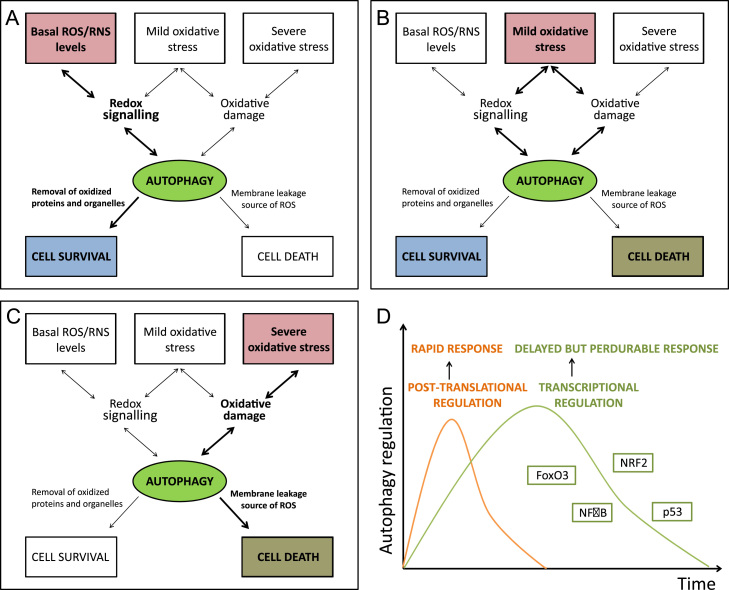
Contribution of autophagy to either cell survival or cell death. Basal ROS levels contribute to cell signaling and also basal autophagy, necessary to maintain protein turnover and proteostasis. A, under mild oxidative stress conditions, redox-mediated signaling and/or oxidative modification of macromolecules may up-regulate the autophagy flux in order to eliminate non-functional and potentially damaging cellular structures, including aggregates and affected organelles. In this context, autophagy would also exert a pro-survival function. B, the contribution of the lysosomal system to either cell survival or cell death depends on a tightly regulated equilibrium of ROS and autophagy. C, under chronic or massive oxidative stress circumstances, lysosomes could negatively contribute to cell fate as a source of ROS, leakage of proteases into the cytosol, degradation of essential cellular components and eventually leading to autophagy cell death. D, the bi-phasic autophagy response to stress. In the first phase there is a rapid increase in the autophagy flux that is mediated by post-translational protein modifications. This response may be followed by a delayed and extended phase that relies on the activation of specific transcriptional programs including NRF2, NFκB, p53 or FoxO3.

Mild oxidative stress conditions, redox-mediated signaling or oxidative modification of macromolecules up-regulate the autophagy flux, leading to elimination of non-functional and potentially damaging protein aggregates and affected organelles (Fig. 5A and B). Several groups have reported redox-modification of autophagy components with a general effect on autophagy induction, although some exceptions can be found (Table 1). Moreover, appropriate GSH levels are essential for basal autophagy. Indeed, GILT (gamma-interferon inducible lysosomal thiol reductase)-deficient fibroblasts show decreased GSH levels and increased autophagy, associated with the up-regulation of the ERK signaling pathway and with nuclear translocation of high-mobility group protein B (HMGB) [49]. Starvation-induced autophagy decreased levels of intracellular GSH, which correlated with increased autophagy flux in different carcinoma cell lines [50].

The importance of redoxtasis for CMA was first reported with the discovery of the degradation of IκB by CMA, as this process was prevented upon treatment with antioxidants [51]. Further studies confirmed that CMA can be activated under mild oxidative stress circumstances and that protein oxidation facilitates the degradation by CMA [52]. Indeed, when known CMA substrates or a pool of cytosolic proteins where incubated with increasing amounts of oxidizing agents, their degradation by CMA was accelerated. One explanation is that oxidative modification of these proteins causes their partial unfolding, not only promoting the exposure of hidden recognition motifs to the chaperones but also facilitating its translocation. Another possibility is that oxidation of certain residues creates a previously inexistent KFERQ-like motif [53]. Increased levels of the CMA mediators LAMP-2A, lys-hsc70, Hip and hsp90 have been reported in this context, suggesting that increased CMA-mediated protein degradation is due to greater binding and up-take of substrates into lysosomes [52]. In accordance with this, CMA down-regulation (through silencing the expression of LAMP-2A) compromised cell viability upon exposure to oxidant and pro-oxidant factors (H_2_O_2_, paraquat or cadmium) [54]. Moreover, the ectopic expression of LAMP-2A in liver of aged rodents (aimed to prevent the age-dependent decrease in CMA) leads to reduced levels of intracellular oxidized and aggregated proteins, improves the response to stressors and preserves organ function [55].

Recently, an oxidative stress-induced form of microautophagy was described, arising from mitochondria-derived vesicles (MDVs) [56,57]. MDVs transported to the lysosomes are enriched in oxidized proteins [58] and are generated in response to oxidative stress [57]. Protein degradation mediated by MDVs does not require activation of the autophagy key actors Atg5, Rab9 or Beclin-1, but it does require the protein kinase PINK1 and the ubiquitin E3 ligase Parkin [59]. This suggests that similar mechanisms apply as in mitophagy, but at restricted patches of the mitochondrial surface. MDVs may thus be generated as a first order of defense to handle redox-damaged proteins in order to prevent functional failure of the organelle.

Apart from an initial and rapid increase in the autophagy flux mediated by post-translational protein modifications, a delayed and extended autophagy response relies on the activation of specific transcription factors such as NRF2, NFκB, p53 or FOXO3a (Fig. 5D) [60]. NRF2, the master regulator of redox homeostasis, has been recently related to the modulation of autophagy. The autophagy-related protein p62/SQSTM1 binds to KEAP1 at the NRF2 binding site, thus promoting NRF2 release from KEAP1 and enabling NRF2-dependent gene expression. Binding of p62 to KEAP1 is favored upon phosphorylation of p62 in an mTORC1 dependent manner [61]. One dimer of p62 can bind to both KEAP1 and LC3, resulting in its degradation. Moreover, the p62 gene has been shown to be a target of NRF2, creating a positive feedback loop where p62 modulates NRF2 protein levels, which in turn control p62 gene expression [62]. Interestingly, NRF2 may modulate other autophagy players as it has been reported to reduce phospho-TAU levels in a mouse model of AD through inducing the expression of NDP-52 [63]. Regarding CMA, the levels of LAMP2A mRNA in the livers of rats treated with paraquat were significantly higher than in untreated rats, although degradation of LAMP2A was only slightly reduced and the distribution between matrix and membrane was not altered [52]. The exact transcription factors involved in this up-regulation have not yet been elucidated, but ROS regulated T-cell receptor-induced LAMP2A expression may rely on RISP, DUOX1 or NFAT activities [64].

The induction of autophagy by ROS can, in turn, modulate ROS levels. Thus, autophagy activation with trehalose was accompanied by the up-regulation of glutathione levels, supporting the antioxidant role of autophagy [65]. Another example is the p53-inducible TIGAR protein, whose ability to inhibit autophagy has been correlated with the suppression of ROS, with no clear effects on the mTOR pathway and is p53 independent [66].

Covalent cross-links, disulfide bonds, hydrophobic interactions and heavily oxidized aggregates are less effectively degraded by the proteasome [67] and are redirected to degradation by the autophagy system [67,68]. If these irreversible aggregates become resistant to hydrolases, they accumulate in the form of lipofuscin [69,70]. Moreover, cross-linking of proteins at the lysosomal membrane can increase proton permeability and luminal pH [71]. As a consequence of this, lysosomal hydrolases are less effective at degrading their substrates, which will favor the accumulation of lipofuscin [69]. Lipofuscin-loaded human fibroblasts exhibit reduced autophagy [72].

Oxidative damage to the lysosomal membrane may lead to the leakage of hydrolases into the cytosol, resulting in further cellular damage (Fig. 5C) [73–75]. The direct damage of the lysosomal membrane by specific oxyproteins, such as ferritin or low density lipoproteins (LDL) in macrophages, has been reported [76]. Moreover, lysosomes themselves can be a source of ROS. Thus, inhibition of autophagy with methylamine, chloroquine or 3-methyladenine prevented ROS formation [77]. Autophagy degradation of catalase under caspase inhibition conditions has also been proposed as a mechanism contributing to the accumulation of ROS [78]. The degradation of iron-containing macromolecules or organelles (ferritin or mitochondrial electron transport complexes for example) leads to the intra-lysosomal accumulation of Fe^2+^. Besides, H_2_O_2_ resulting from different cellular processes can either enter the lysosome or be generated in its lumen as a result of degraded mitochondria. Both species may react (Fenton reaction), resulting in the formation of the extremely reactive hydroxyl radicals [79].

## 4 The unfolded protein response (UPR) in management of oxyproteins

The UPR is an adaptive response to ER stress [80] meant to reduce the protein folding load in the ER and increase ER folding capacity [81]. This is achieved by a general suppression of translation, retrotranslocation of proteins and their degradation by ERAD (ER associated protein degradation), degradation of ER-associated mRNAs, expansion in ER volume and increased synthesis of ER chaperones (reviewed in [81]). Oxidative stress leads to protein unfolding or misfolding in the endoplasmic reticulum (ER) which in turn produce ER stress and activate the UPR [82].

Three membrane-associated proteins have been identified for sensing ER stress in eukaryotes: activating transcription factor 6 (ATF6), pancreatic ER eIF2α kinase (PERK, also double-stranded RNA-activated protein kinase-like ER kinase), and inositol-requiring kinase 1 (IRE1) [83]. The luminal domain of each sensor is bound to the chaperone 78 kDa glucose-regulated protein (GRP78/BiP) in the resting state. GRP78/BiP dissociates upon ER stress to bind unfolded proteins, leading to the activation of the three sensors [83,84].

Upon release from GRP78/BiP, ATF6 reaches the Golgi apparatus, where it is sequentially cleaved and the fragment generated (ATF6f) is capable to enter the nucleus and induce transcription of UPR genes that collectively may reduce ER stress, such as XBPI, BiP or CHOP [84–88]. ATF6 contains two conserved Cys residues that can form intra- and intermolecular disulfide bonds. Upon ER stress, only the reduced monomer form can reach the Golgi apparatus to be cleaved and act as a transcription factor, and therefore redox control of the disulfide bonds in ATF6 is crucial for its export [89].

**Fig. 6:**
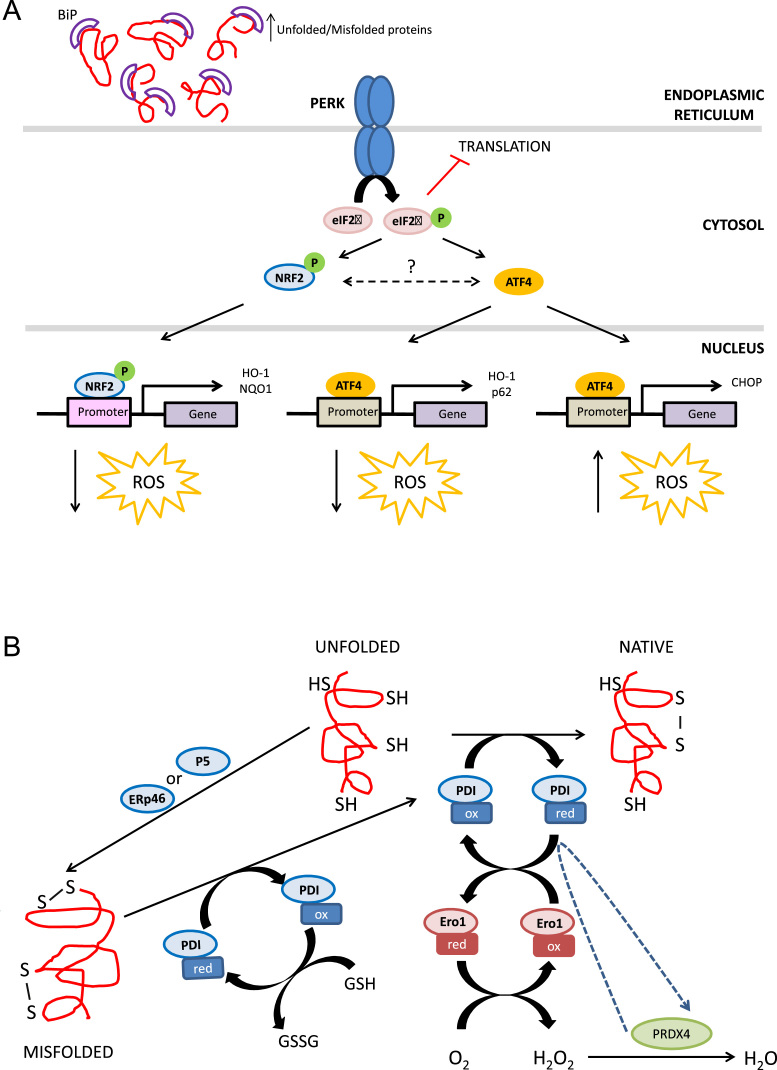
Impact of protein folding on oxidative stress and the UPR. A, Oxidative environment in the ER is crucial for correct protein folding. The highly oxidative environment in the ER (1:1–1:3 GSH/GSSH ratio compared to the 30:1–100:1 in the cytosol) is required for protein folding; ROS are formed as byproducts of normal protein folding and as a consequence of incorrect disulfide bond formation. Protein disulfide isomerases (PDIs) catalize disulfide bond formation in nascent proteins, a process necessary for correct protein folding. Two Cys in the active site of PDI accept two electrons from the Cys of the folding polypeptide. PDIs are then oxidized by oxidoreductin 1 (ERO1) proteins that subsequently transfer electrons to oxygen to produce H_2_O_2_. The generated H_2_O_2_ is metabolized into H_2_O by peroxiredoxin IV. Moreover, improperly paired disulfide bonds can be reduced by PDIs in parallel with glutathione oxidation. ROS produced during protein folding and the consumption of GSH upon the reduction of improperly paired disulfide bonds may shift the redox balance in the ER, favoring the accumulation of misfolded/unfolded proteins and activating the UPR. B, The UPR modulates redoxstasis. One of the three UPR arms activated upon ER stress is PERK signaling. PERK phosphorylates NRF2, promoting its nuclear translocation and induction of the antioxidant response. PERK signaling also involves phosphorylation of eIF2α and a transient inhibition of protein translation. However, selective translation of the transcription factor ATF4 is induced under these circumstances. ATF4 promotes an antioxidant response through the expression of target genes such as HO-1. Although a crosstalk between ATF4 and NRF2 has been described, the underlying mechanisms remain unclear. ATF4-induced CHOP expression results in the expression of Ero1α, an enzyme that causes Ca^2+^ leakage from the ER, activating CaMKII in the cytosol, which in turn induces NOX2 and causes oxidative stress. Moreover, CHOP has been implicated in the transcriptional repression of Bcl2 and transactivation of BIM and PUMA that leads to enhanced oxidant injury and apoptosis.

Most of the PERK signaling is mediated through phosphorylation of the alpha subunit of the eukaryotic initiation translation factor 2 (eIF2α), which transiently inhibits protein translation. However, selective translation of the transcription factor ATF4 and subsequent expression of its target genes is induced under these circumstances (Fig. 6A) [86]. ATF4 induces expression of genes like HO-1 and p62/SQSTM1 that are known to be induced upon oxidative stress and can have a role in the modulation of redoxtasis. Moreover, ATF4-induced CHOP expression results in the expression of Ero1α, an enzyme that contributes to oxidation in the ER (Fig. 6A) [90]. Ero1α causes Ca^2+^ leakage from the ER, activating CaMKII in the cytosol, which in turn induces NOX2 and causes oxidative stress. Moreover, in cooperation with FoxO3, CHOP has been implicated in the transcriptional repression of Bcl2 and transactivation of BIM and PUMA that leads to enhanced oxidant injury and apoptosis (Fig. 6A) [91,92].

PERK phosphorylates NRF2 promoting its dissociation from KEAP1, translocation to the nucleus and expression of its target genes [93,94]. Different UPR genes have been reported to contain AREs in their promoter regions [95]. Since NRF2 also promotes the expression of proteasome and autophagy-genes it is not surprising that tunicamycin-induced ER stress significantly up-regulates proteasomal activity [96].

The transcription factor NRF1, also involved in the antioxidant response, localizes to the ER membrane and may undergo nuclear translocation upon deglycosilation or intra-membrane/proteasomal cleavage. NRF1 localization along with its ability to up-regulate proteasomal subunits after proteasome inhibition makes it reasonable to consider an NRF1 up-regulatory function of ERAD during ER stress. Tunicamycin treatment of myc-tagged NRF1 transfected cells resulted in higher levels of the 110 kDa Nrf1 fragment in the nucleus when compared with untreated cells. However, another group later reported that this isoform does not have the ability to transactivate ARE-containing genes [96].

Activated IRE1 contains both a kinase and a RNAse domain. Its activation leads to mRNA splicing of an intron from transcription factor X box binding protein 1 (XBP-1) to trigger its translation [80]. XBP-1 modulates expression of UPR target genes, including ER chaperons, glycosylation enzymes or ERAD components [97]. Other RNAs are targeted through a process called regulated IRE1-dependent decay (RIDD) that reduces the amount of proteins in the ER. IRE1 kinase domain leads to the activation of the IRE1-TRAF2-JNK axis. Activation of NRF2 by MAPK is a controversial issue that appears to be context-dependent. However, in JNK-activated ER stress, the inhibition of JNK leads to NRF2 over-activation [96]. These results point to NRF2 as a potential substrate of the IRE1-TRAF2-JNK pathway.

The ER environment is highly oxidative because the ratio GSH/GSSH is 1:1–1:3 compared to 30:1–100:1 in the cytosol [83]. This environment favors the formation of disulfide bonds necessary for correct protein folding in an ezymatic reaction catalized by protein disulfide isomerases (PDIs) (Fig. 6B) [98]. These enzymes also reduce improperly formed disulfide bonds in a process accompanied by glutathione consumption. Most PDIs contain at least one thioredoxin (Trx)-like catalytic domain. PDIs are oxidized by oxidoreductin proteins 1 (Ero1α and Ero1β in mammals), which regenerate themselves by transfering electrons to oxygen to produce H_2_O_2_ [98,99]. Peroxiredoxin IV metabolizes H_2_O_2_ into H_2_O [100]. Disruption of redox balance in the ER can result in incorrect disulfide bond formation during protein folding, redox imbalance and oxidative stress [101].

ROS production and oxidative stress can be considered an integral component of the UPR, triggering both transcriptional and post-translational responses which in turn lead to cell adaptation and survival or cell death by apoptosis.

## 5 Neurodegenerative diseases as models of oxyprotein pathology

### 5.1 Prion diseases

Although the physiological role of mammalian prion protein (PrPc) is not known, there is some evidence suggesting that it could play a role as endogenous ROS scavenger, protecting other structural and signaling proteins, because it has a high number of sulfhydryl groups in methionine residues [102–104]. In addition, PrPc could be a redox switch since methionine sulfoxidation is involved in cell signaling [105].With ageing, there is a progressive accumulation of oxidized methionine residues in PrPc that contribute to protein misfolding, and participate in the transition from a monomeric globular form with α-helical content to a self-aggregating form with extended β-strand-rich structure with “infective” capacity to transmit the corrupted conformation to other native prion proteins [106,107]. The transition of PrPc N-terminal region, from a random coil to a β-sheet structure transforms the soluble and protease-sensitive PrPc into the oxidized, insoluble and relatively protease-resistant PrPsc [108,109]. PrPsc monomers and small oligomers induce nerve cell death after internalization and accumulation into the endolysosomal compartment where they cause lysosomal damage with subsequent proteolytic enzyme leakage and activation of caspase-dependent apoptosis [110,111]. Autophagy efficiency is also attenuated, due to PrPsc protease-resistance, and aggregation of misfolded PrPsc also leads to mitochondrial failure [112–114]. Assuming that the primary role of PrPc is to act as a global cell antioxidant, regulating the oxidative state of structural and signaling proteins, the conversion of soluble PrPc proteins to less soluble and aggregation prone oxidized PrPsc proteins could lead to a major depletion of the antioxidant PrPc pool thus leading to neuronal death.

### 5.2 Alzheimer's Disease (AD)

The key pathological features of Alzheimer’s disease are the presence of soluble amyloid β-peptides, for example Aβ(1–42), that accumulate in the intracellular and extracellular space and neurofibrillary tangles made of TAU protein aggregates. The amyloid β-peptides can be further modified into misfolded Aβ monomers, dimers, oligomers and intermediate products that are toxic, leading to cell death [115,116]. The oxidation of Aβ(1–42) at the 35 methionine residue promoted by for example H_2_O_2_ or Cu^2+^, accelerates the production of toxic Aβ(1–42) products and further protein oxidation and lipid peroxidation [117]. Compared to reduced Aβ(1–42), oxidized Aβ(1–42) is more resistant to degradation by autophagy and endosome–lysosome fusion which further contributes to its accumulation and toxic effects due to release of undigested contents into the cytosol [118–121]. The toxicity of amyloid β-peptide derivatives decreases with protofibril and fibril formation and terminates in the formation of stable and inert amyloid plaques [115,116]. Increased ROS formation, inside or outside the cell, favors the transition of Aβ monomers into toxic forms and also stimulates the breakdown of microtubule cytoskeleton by promoting zinc- or H_2_O_2_-induced TAU phosphorylation [116,122]. Hyperphosphorylated TAU, together with the oxidized form of Aβ(1–42), leads to mitochondrial damage, subsequent reduction in ATP production, decreased mitochondrial potential, production of more ROS and finally cell death [123].

Monomeric TAU is a natively unfolded and short-lived protein, thus being a good substrate for the 20S proteasome under normal conditions [124]. Under stressing conditions it has been reported that the E3 ligase CHIP (Carboxy terminus Hsp70 interacting Protein), participates in TAU ubiquitlation leading to its degradation by the 26S proteasome [125]. However, as indicated before, the 26S proteasome is sensitive to oxidative stress and loses efficacy in degradation of TAU, particularly when it has undergone posttranslational modifications (phosphorylation and oxidation among other modifications) and led to formation of toxic oligomers and aggregation. In fact, it has been reported that the proteasome activity is impaired in AD brains [126] and that TAU-containing paired helical filaments inhibit the proteasome [127]. Under these conditions TAU is degraded by p62-driven macroautophagy and LAMP2A-driven chaperone mediated autophagy [128]. Of note, chaperone mediated autophagy is up-regulated under oxidative stress conditions, further suggesting its role in degradation of oxidized and aggregated TAU [52]. A new mechanism of redox-dependent degradation of TAU has been described [63]. The gene coding the autophagy adapter protein NDP52, contains several antioxidant response elements regulated by NRF2. In fact, in *Nrf2*-knockout mice, phosphorylated TAU accumulates in the brains concurrent with decreased levels of NDP52. In summary, TAU is a substrate of both the proteasome and the autophagy systems and both processes appear to be impaired in AD [129].

### 5.3 Parkinson's disease

α-Synuclein (α-SYN) is localized to presynaptic terminals in the central nervous system and modulates vesicular release of dopamine [130–132] by attenuating its release under circumstances of repeated firing [133]. *In vivo*, α-SYN can be present as a soluble unfolded protein that can aggregate into progressively less soluble oligomers, protofibrils and insoluble amyloid fibril form [134]. This protein is normally degraded through the UPS, macroautophagy or CMA; but nitration and oxidative modifications slow down or even inhibit its proteasomal degradation. Besides, partial degradation can lead to its C-terminal truncation, favoring further aggregation. In addition, α-syn contains a KFERQ consensus sequence and oxidation of this protein (for instance, dopamine-modified α-syn) prevents its translocation into the lysosome lumen, acting as uptake blockers and preventing degradation of other CMA substrates. Attenuation of chaperone-mediated autophagy degradation leads to a compensatory increase in macroautophagy, accumulation of autophagosomes, and ultimately to cell death due to the release of undigested contents into the cytosol [135]. Posttranslational modification of α-SYN, for example by phosphorylation, ubiquitination, nitration or oxidation, also reduces autophagosome degradation and promotes α-SYN oligomerisation. The toxic effect of these posttranslational modifications can be compounded by the propensity of α-SYN to bind to various molecules in neuronal cells [136]. For example, Fe2+, dopamine or H_2_O_2_ oxidize methionine residues in the α-SYN monomer, that is the predominant form of oxidized α-SYN, and also promote the formation of stable oligomers with a resistance to fibrilisation that is proportional to the number of oxidized methionine residues [137]. The oxidized α-SYN monomer can interact with lipids, as well as with proteins, changing their redox state and function thus sharing similarities with the oxidized Aβ(1–42) [138]. For example, the oxidized α-SYN monomer disrupts autophagy and also disrupts mitochondrial function [139–141].

Recently, it has been shown that the Parkinson's associated protein DJ-1 binds to and inhibits the 20S proteasome and thus prevents the degradation of substrates such as a-syn or p53. Moreover, under oxidative stress conditions, DJ-1 induces the NRF2-dependent antioxidant response. Among the induced genes, NQO1 reinforces DJ-1 function by also inhibiting the 20S proteasome. However, NRF2 also induces the expression of 20S proteasome subunits. This robust regulation may be necessary to maintain proper 20S proteasome activity [142].

## 6 Future perspectives

Oxidative modification of proteins and proteolytic pathways compromise protein quality and cell viability. These events may be among the most relevant in driving protein toxicity in several pathologies as exemplified here for neurodegenerative diseases. It is now essential to find ways to prevent these effects through either reinforcing redox homeostasis or increasing the capacity of proteolytic systems.

## Figures and Tables

**Table 1:** Redox-modification and effects of autophagy components.

**Stimulus/treatment**	**Oxidative modified target**	**Consequences in autophagy**	**Ref.**
Starvation-induced ROS (H_2_O_2_)	Atg4 (inhibition)	Atg4 cannot delipidate LC3, increased autophagy	[143]
Oxidative stress (H_2_O_2,_ mitocondrial ROS), hypoxia-induced mtROS	AMPK (activation)	AMPK inhibits mTORC1 and activates ULK1, increased autophagy	[144–146]
Genotoxic and oxidative stress	ATM (activation)	Activation of AMPK and TSC2, increased autophagy	[141–147]
Oxidative stress	Trx (oxidation and dissociation from ASK1)	Activation of ASK1, which activates JNK1 promoting release of Beclin 1 and autophagy induction	[148–150]
S-nitrosylation	JNK1, IKKβ (inhibition)	Decreased phosphorylation of AMPK and TSC2, decreased autophagy	[150,151]
NO	JNK (inhibition)	Bcl-2 phosphorylation, decreased Bcl2-Beclin1 complex, increased autophagy	[150,151]
Oxidative stress (H_2_O_2_), low NO levels	PTEN (inhibition)	PI3K/Akt activation, phosphorylation of Beclin-1 and activation of the mTOR complex, autophagy inhibition	[150,152]
High NO levels	Akt (inhibition)	Reduced phosphorylation of Beclin-1 and inactivation of the mTOR complex, increased autophagy	[150,152,153]
Sulfhydration	PARKIN (activation)	Increased localization to the mitochondrial membrane, increased mitophagy	[154]
O-GlcNAcylation	BCL2, BECLIN-1	Inhibition of autophagy	[154]
O-GlcNAcylation	AMPK (activation)	AMPK inhibits mTORC1 and activates ULK1, increased autophagy	[155]
